# Primary Chest Wall Glomus Tumor Resected by Video-Assisted Thoracoscopic Surgery: A Case Report with 10-Year Follow-up

**DOI:** 10.70352/scrj.cr.26-0226

**Published:** 2026-07-24

**Authors:** Noritake Kikunishi, Tetsukan Woo, Daisuke Noma, Atsuko Osawa, Takashi Nakayama, Aya Saito

**Affiliations:** 1 Department of Surgery, Yokohama City University School of Medicine, Yokohama, Kanagawa, Japan; 2 Department of Thoracic Surgery, Saiseikai Yokohama City Nanbu Hospital, Yokohama, Kanagawa, Japan; 3 Department of Pathology, Saiseikai Yokohama City Nanbu Hospital, Yokohama, Kanagawa, Japan

**Keywords:** glomus tumor, chest wall, video-assisted thoracoscopic surgery, FDG-PET/CT, back pain, perivascular tumor

## Abstract

**INTRODUCTION:**

Glomus tumors are rare perivascular neoplasms that typically occur in the distal extremities. Their occurrence in the chest wall is extremely rare, with only 13 cases previously reported in the English literature. Herein, we report a unique case of a primary chest wall glomus tumor in a patient who presented with severe, rapidly worsening back pain, underwent ^18^F-fluorodeoxyglucose-PET/CT (FDG-PET/CT) evaluation, and experienced complete pain resolution following thoracoscopic resection.

**CASE PRESENTATION:**

A 65-year-old man presented to our emergency department with a 1-month history of progressively worsening right posterior back pain. Contrast-enhanced CT revealed a 20 × 13-mm well-enhanced oval mass in the right posterior chest wall at the level of the ninth rib. MRI demonstrated heterogeneous intensity on T2-weighted images. FDG-PET/CT showed low tumor uptake with a maximum standardized uptake value (SUVmax) of 2.2. This finding was considered adjunctive metabolic information that did not strongly suggest a highly aggressive malignancy; however, it was insufficient to exclude malignancy or establish a definitive diagnosis. To achieve a definitive diagnosis and alleviate the severe pain, surgical resection was performed via 4-port video-assisted thoracoscopic surgery (VATS). The tumor was completely excised with clear margins. Histopathological and immunohistochemical analyses confirmed the diagnosis of a benign glomus tumor, with diffuse positivity for smooth muscle actin (SMA) and a Ki-67 labeling index of less than 1%. The patient’s severe back pain completely resolved immediately after surgery, and he remained completely asymptomatic with no evidence of recurrence at the 10-year follow-up.

**CONCLUSIONS:**

Although exceedingly rare, glomus tumors should be included in the differential diagnosis of painful chest wall tumors. FDG-PET/CT may provide adjunctive information regarding metabolic activity during preoperative assessment; however, its findings should be interpreted cautiously and cannot replace histopathological evaluation. To the best of our knowledge, this is the first reported case of a chest wall glomus tumor evaluated with FDG-PET/CT. Complete surgical resection remains essential for definitive diagnosis and symptom relief, and minimally invasive resection via VATS may be an effective approach when complete resection is feasible.

## Abbreviations

FDG-PET/CT
: ^18^F-fluorodeoxyglucose PET/CT
H&E
: hematoxylin and eosin
NRS
: numerical rating scale
SMA
: smooth muscle actin
SUVmax
: maximum standardized uptake value
VAS
: visual analogue scale
VATS
: video-assisted thoracoscopic surgery

## INTRODUCTION

Glomus tumors are rare perivascular neoplasms arising from the neuromyoarterial glomus body, which functions as a specialized form of arteriovenous anastomosis involved in thermoregulation. They account for approximately 1%–2% of soft tissue tumors and typically manifest in the subungual region of the hands. According to the latest World Health Organization (WHO) Classification of Tumours (5th edition, 2020), glomus tumors are categorized within the group of pericytic (perivascular) tumors.^1)^

The occurrence of glomus tumors in the chest wall is exceedingly rare. To date, only 13 cases have been reported in the English literature.^2–14)^ Due to their rarity, deep-seated location, and lack of the classic clinical triad (localized pain, pinpoint tenderness, and cold hypersensitivity), these tumors are often misdiagnosed as other mesenchymal tumors such as schwannomas or hemangiomas,^14,15)^ leading to delayed diagnosis and prolonged patient suffering.

Herein, we report a unique case of a primary chest wall glomus tumor presenting with severe, rapidly worsening back pain that necessitated emergency department evaluation. This case is notable for 3 reasons: (1) it is, to the best of our knowledge, the first reported chest wall glomus tumor evaluated with FDG-PET/CT; (2) the pain completely resolved immediately after surgery following minimally invasive resection; and (3) the tumor size of 2.0 cm with deep location satisfied the criteria for uncertain malignant potential, yet was confirmed benign by a very low Ki-67 index (<1%) and absence of nuclear atypia.

## CASE PRESENTATION

A 65-year-old man had been experiencing right posterior back pain for 1 month. The pain was somatic in character, without positional dependency, respiratory variation, or neurological symptoms, and progressively worsened to a severity requiring emergency department evaluation. He had no relevant past medical history. Physical examination revealed no skin lesions, no palpable mass, and no tenderness on the chest wall. Laboratory findings were unremarkable.

**Fig. 1:**
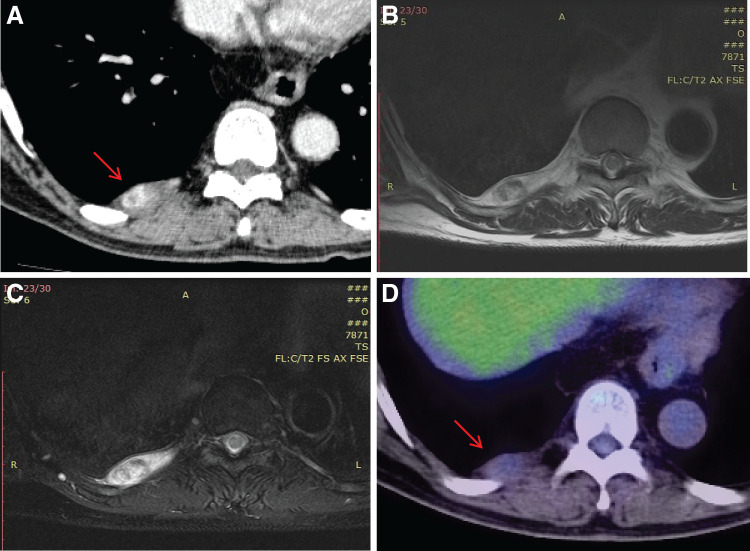
Radiological findings of the chest wall tumor. (**A**) Contrast-enhanced CT scan showing a well-circumscribed, heterogeneously enhanced oval mass in the right posterior chest wall at the level of the ninth rib (arrow). (**B**) T2-weighted MRI revealing heterogeneous high signal intensity in the tumor. (**C**) Fat-suppressed T2-weighted MRI clearly delineating the tumor from the surrounding fatty tissue. (**D**) FDG-PET/CT fusion image showing mild accumulation in the tumor with a SUVmax of 2.2 (arrow). FDG-PET/CT ^18^F-fluorodeoxyglucose PET/CT; SUVmax, maximum standardized uptake value

Contrast-enhanced CT demonstrated a 20 × 13-mm oval-shaped mass in the right posterior chest wall at the level of the ninth rib, exhibiting heterogeneous density with strong enhancement (**Fig. 1A**). MRI showed heterogeneous intensity on T2-weighted images (**Fig. 1B**) and fat-suppressed T2-weighted MRI clearly delineating the tumor from the surrounding fatty tissue (**Fig. 1C**). FDG-PET/CT demonstrated weak uptake in the tumor, with an SUVmax of 2.2 (**Fig. 1D**). These imaging findings were suggestive of a benign or low-grade vascular or neurogenic tumor; however, a definitive preoperative diagnosis could not be established, as the differential diagnosis included a broad range of entities such as schwannoma, hemangioma, solitary fibrous tumor, angioleiomyoma, pericytic tumors, and low-grade sarcomas.

**Fig. 2:**
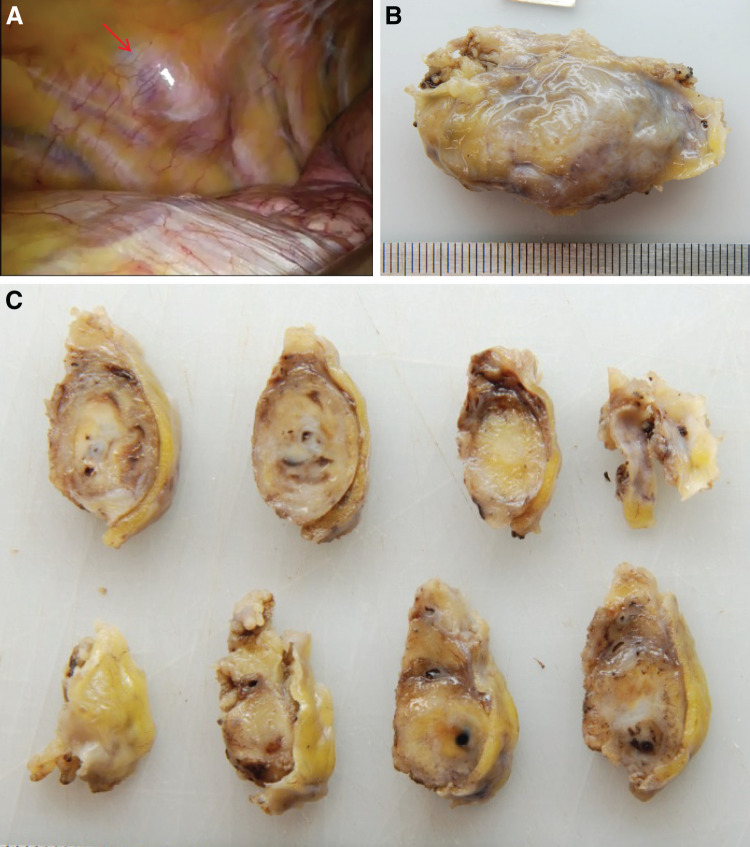
Intraoperative and macroscopic findings. (**A**) Thoracoscopic view showing a soft, circumscribed, and highly vascular tumor (arrow) adherent to the parietal pleura. (**B**, **C**) Macroscopic appearance of the resected specimen showing a heterogeneous cut surface with white to brown areas and focal hemorrhages.

For definitive diagnosis and treatment, surgical resection was performed via 4-port VATS with the patient in the left lateral decubitus position. A 10-mm camera port was placed in the eighth intercostal space at the midaxillary line. Three additional 5-mm working ports were placed in the fifth intercostal space at the anterior axillary line, the sixth intercostal space at the posterior axillary line, and the seventh intercostal space at the anterior axillary line. The tumor was identified as a soft, circumscribed, and highly vascular mass located in the extrapleural space anterior to the intercostal muscles in the right posterior chest wall at the level of the ninth rib, adherent to the parietal pleura, without evident attachment to the intercostal neurovascular bundle or periosteum based on intraoperative findings (**Fig. 2A**). The precise site of origin could not be determined, but the tumor was considered to arise from the extrapleural soft tissue. It was completely resected en bloc with the overlying parietal pleura, achieving clear resection margins using endoscopic scissors and bipolar coagulation forceps. Pathological examination confirmed microscopically negative margins, although the exact margin distance was not measured. Estimated blood loss was minimal, and no rib resection was required.

**Fig. 3:**
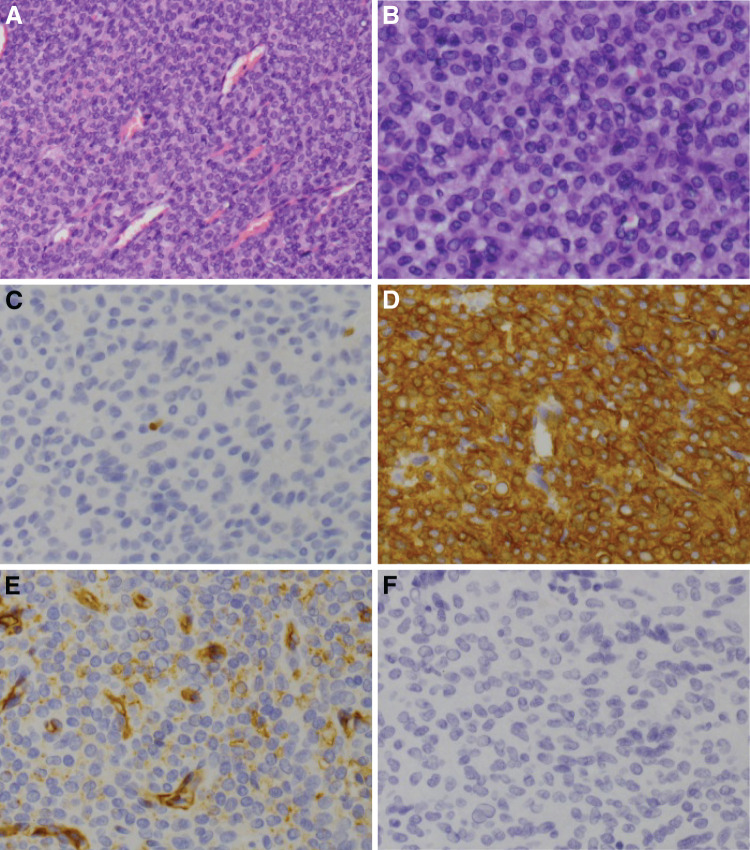
Histopathological and immunohistochemical findings. (**A**, **B**) H&E staining showing the tumor composed of nests of small, uniform, rounded cells with centrally placed round nuclei and amphophilic to lightly eosinophilic cytoplasm. The tumor cells are arranged around abundant small blood vessels in a solid or sheet-like pattern (**A**, ×100; **B**, ×200). (**C**) The Ki-67 labeling index was less than 1% (×200). (**D**) Immunohistochemical staining showing diffuse positivity for SMA (×200). (**E**) Negative staining for CD34 (×200). (**F**) Negative staining for S-100 protein (×200). H&E, hematoxylin and eosin; SMA, smooth muscle actin

Macroscopically, the cut surfaces of the tumor were heterogeneous, white to brown in color, with focal hemorrhages (**Fig. 2B** and **2C**). Histologically, the tumor consisted of solid or sheet-like nests of small, uniform, rounded cells with centrally placed round nuclei and amphophilic to lightly eosinophilic cytoplasm, surrounding abundant small blood vessels (**Fig. 3A** and **3B**). Neither marked nuclear atypia nor atypical mitotic figures were observed, and the Ki-67 labeling index was less than 1% (**Fig. 3C**). Immunohistochemical analysis revealed that the tumor cells were diffusely positive for SMA (**Fig. 3D**), but negative for CD34, S-100, HMB-45, desmin, epithelial membrane antigen, AE1/3, melan-A, and calretinin (**Fig. 3E** and **3F**). These findings confirmed the diagnosis of a benign glomus tumor of the chest wall.^1,16)^

The patient’s severe back pain completely resolved immediately after surgery. Although formal pain scores (numerical rating scale [NRS]/visual analogue scale [VAS]) were not prospectively recorded, no additional analgesics beyond routine postoperative loxoprofen were required. The patient reported complete resolution of the deep posterior back pain that had prompted emergency department evaluation, which was clearly distinguishable from expected postoperative wound pain at the port sites. He had an uneventful postoperative recovery and was discharged 1 week after surgery. The patient has been followed up annually with blood tests, chest radiography, and CT. At the last follow-up in February 2026, 10 years after surgery, he remained completely asymptomatic with no clinical or radiological evidence of recurrence.

## DISCUSSION

Glomus tumors of the chest wall are exceedingly rare mesenchymal neoplasms. We performed a literature search of PubMed and Google Scholar using the following terms: “glomus tumor,” “glomangioma,” “glomangiomyoma,” “chest wall,” “thoracic wall,” and “intercostal.” The last search was performed in March 2026. We included cases of primary glomus tumors arising from the chest wall, including cutaneous, subcutaneous, intramuscular, intercostal, and subpleural lesions. Cases arising primarily from the lung, mediastinum, or visceral pleura without clear chest wall origin were excluded. English-language articles and non-English articles with sufficient English abstracts were reviewed. Including the present case, 14 cases met these criteria and were summarized in **Table 1**.^2–14)^ Among these, the male-to-female ratio was 10:3, and the mean age at diagnosis was approximately 46 years (range, 19–67 years). The mean symptom duration before diagnosis was notably long (5.4 years),^14)^ reflecting the diagnostic challenges posed by this entity. In the present case, however, the pain rapidly worsened over 1 month and prompted emergency department evaluation, a clinical course more acute than that of most previously reported cases.

**Table 1:** Comparison of reported cases of glomus tumors of the chest wall

Author (year)	Sex	Age (years)	Symptom duration	Chief symptom	Skin involvement	Location	Size (cm)	Preoperative imaging	Histological subtype	Surgical approach	Benign/malignant	Outcome (follow-up)
Schneller^2)^ (2001)	M	30	10 years	Pain	NR	Multifocal; left posterior intercostal spaces	Up to 9 (multiple)	CT	Glomangiomyoma	Open resection	Benign (multifocal)	NR
Tsuruta et al.^3)†^ (2003)	F	19	5 years	Pain	No	Right anterior, 3rd intercostal space	2.0	US, CT, MRI: T2 hyperintense	Solid glomus tumor	Open resection	Uncertain malignant potential^‡^	No recurrence (1 year)
Neelaiah and Suryanarayanarao^4)^ (2005)	M	46	2 years	Pain	Bluish-red nodule	Subcutaneous anterior chest wall	0.5 × 0.75	NR	Solid glomus tumor	NR	NR	NR
Uchiyama et al.^5)†^ (2011)	M	50	10 years	Pain	NR	Right 3rd intercostal space (+ buttocks)	NR	CT	Solid glomus tumor	Resection (incl. 4th rib)	Uncertain malignant potential^‡^	NR
Venugopal^6)^ (2015)	M	67	6 years	Pain, cold sensitivity	Vague swelling	Left scapula area	2.0	NR	Solid glomus tumor	Open excision	Benign	NR
Temiz et al.^7)^ (2016)	NR	NR	NR	Pain	Purple-colored hard nodule	Sternal projection (subcutaneous)	0.5–1.0	US	Solid glomus tumor	Open excision	Benign	No recurrence (1 year)
Yim et al.^8)^ (2016)	M	41	NR	Pain	NR	Right paraspinal area	6.3 (->19 at recurrence)	MRI	Solid glomus tumor	Open resection × 2	Malignant transformation at recurrence	Recurrence at 6 months and 4 years
Kambhampati and Kambhampati^9)^ (2018)	M	47	2 years	Pain	Painful purple papule	Left anterior chest wall (subcutaneous)	1.0 × 0.4	US	Solid glomus tumor	Open excision	Benign	No recurrence (6 months)
Zanjani et al.^10)^ (2021)	M	63	15 years	Pain, cold sensitivity, limited shoulder movement	Axillary lump	Left lateral chest wall	5.0	MRI: T2 hyperintense	Glomangioma	Open resection	Benign	No recurrence (19 months)
Alyaseen et al.^11)^ (2021)	M	35	7 years	Pain, cold sensitivity	Bluish papule	Right lateral chest wall (cutaneous)	1.0	NR	Glomangioma	Open excision	Benign	NR
Bhalchandra Londhe et al.^12)^ (2021)	F	27	NR	Pain (scapulothoracic impingement)	No	Right posterior chest wall muscles	2.0 × 1.0 × 1.9	MRI: T2 hyperintense	Solid glomus tumor	Open resection	Benign	No recurrence (1 year)
Engle et al.^13)^ (2024)	F	67	6 months	NR	Skin-colored subcutaneous nodule	Anterior chest wall (+ lung metastases)	NR	CT	Solid glomus tumor	Radiation + pembrolizumab	Malignant (lung metastases)	Partial remission
Jagelavicius et al.^14)^ (2025)	M	42	1 year	Intermittent pain under right scapula	No	Right posterior chest wall, 9th intercostal space (subpleural)	3.8 × 1.8	CT: well-defined; MRI: T1 hyperintense, T2 isointense, contrast-enhanced	Glomangioma	VATS	Benign	No recurrence (3 months)
**Present case**	M	65	1 month (rapidly worsening; ER visit)	Severe right posterior back pain	No	Right posterior chest wall, 9th rib level (deep-seated)	2.0 × 1.3	CT: heterogeneous strong enhancement; MRI: T2 heterogeneous; FDG-PET: SUVmax 2.2	Solid glomus tumor	4-port VATS	Benign (Ki-67 <1%, no atypia)	Complete pain resolution immediately after surgery; no recurrence (10 years)

Bold row indicates the present case.

^†^Only abstract available in English.

^‡^Classified as uncertain malignant potential based on deep location and size ≥2 cm per Folpe criteria^16)^; full pathological details not reported.

ER, emergency room; FDG-PET/CT, ^18^F-fluorodeoxyglucose PET/CT; NR, not reported; SUVmax, maximum standardized uptake value; VATS, video-assisted thoracoscopic surgery

Reported chest wall glomus tumors can be broadly divided into superficial lesions involving the skin or subcutaneous tissue and deeply located lesions arising from the intercostal, muscular, or subpleural chest wall. Superficial lesions often present with visible skin discoloration, a palpable painful nodule, or cold sensitivity, whereas deeply located lesions may lack the classic triad and present only with nonspecific deep chest wall or back pain. The present case belonged to the deeply located group, as no skin lesion or palpable mass was observed and the tumor was detected only by cross-sectional imaging.

When the tumor is located in deeper layers, as in the present case, nonspecific deep pain is the predominant symptom, and the classic clinical triad—localized pain, pinpoint tenderness, and cold hypersensitivity—is frequently absent.^1,16)^ The intense pain characteristic of glomus tumors is attributed to the rich innervation of the glomus body and the release of neuropeptides, including substance P, from unmyelinated nerve fibers in and around the tumor.^17,18)^ This neurochemical mechanism likely explains the dramatic and immediate resolution of pain following complete tumor excision observed in the present case and in several previously reported cases.^4,10,12)^ The mechanism underlying the rapid clinical deterioration in the present case remains speculative; however, the focal hemorrhagic areas identified macroscopically suggest that intratumoral hemorrhage may have acutely increased local pressure on the richly innervated glomus body, thereby contributing to the acute exacerbation of pain.

Preoperative imaging is helpful but rarely diagnostic for chest wall glomus tumors. On MRI, glomus tumors classically appear hyperintense on T2-weighted images due to their high vascular content.^18)^ In the present case, the tumor showed heterogeneous T2 signal intensity, which may reflect the focal hemorrhagic areas observed macroscopically. To our knowledge, FDG-PET/CT has not been previously reported in any case of chest wall glomus tumor. In our patient, the low SUVmax of 2.2 was considered adjunctive metabolic information that did not strongly suggest a highly aggressive malignancy; however, it was insufficient to exclude malignancy or establish a definitive diagnosis. The decision to proceed with minimally invasive diagnostic and therapeutic resection was based on the overall clinical context, including the small, well-circumscribed tumor, the absence of radiological evidence of invasion, and the need for both definitive diagnosis and pain relief. Benign neurogenic tumors such as schwannomas may frequently exhibit high FDG uptake, whereas some low-grade sarcomas may show low accumulation. There is currently no established SUVmax threshold or high-level evidence for using PET/CT to determine the surgical approach for glomus tumors. The definitive confirmation of benign nature relied on the Ki-67 labeling index of less than 1% and the absence of nuclear atypia, rather than the preoperative imaging findings alone. These findings suggest that FDG-PET/CT may serve as supportive data in the preoperative workup of deep-seated chest wall tumors of uncertain etiology, while acknowledging that its role remains supplementary rather than definitive.

Regarding malignant potential, Folpe et al. and the WHO classification propose that a glomus tumor is considered malignant if it is both deeply located and larger than 2 cm, while it is considered to have uncertain malignant potential if it meets only one of these criteria, or shows high mitotic activity.^1,16)^ In the present case, the tumor was deeply located, thereby satisfying the criterion for uncertain malignant potential. However, the complete absence of nuclear atypia, absence of atypical mitotic figures, and an extremely low Ki-67 labeling index (<1%) confirmed its benign nature. This case highlights the importance of thorough histopathological evaluation, including Ki-67 assessment, in deep-seated chest wall tumors.

Complete surgical resection is generally accepted as the mainstay for both diagnosis and treatment of glomus tumors.^14)^ Incomplete resection has been associated with recurrence rates of 12%–33%.^14,16)^ In the present case, 4-port VATS enabled complete tumor excision with clear margins and minimal invasiveness, without requiring rib resection. VATS has now been reported in 2 chest wall glomus tumor cases, including ours,^14)^ and may be an effective approach for selected posteriorly located, deep-seated tumors when complete resection is feasible.

## CONCLUSIONS

Although exceedingly rare, glomus tumors should be included in the differential diagnosis of painful chest wall masses, even when the clinical presentation is acute. FDG-PET/CT may provide adjunctive information regarding metabolic activity during preoperative assessment; however, its findings should be interpreted cautiously and cannot replace histopathological evaluation. Complete surgical resection remains essential for definitive diagnosis and symptom relief, and minimally invasive resection via VATS may be an effective approach when complete resection is feasible.

## DECLARATIONS

### Use of artificial intelligence (AI) tools

During the preparation of this manuscript, the authors used Gemini to improve the English language and readability. After using this tool, the authors reviewed and edited the content as needed and take full responsibility for the content of the publication.

### Availability of data and materials

The dataset supporting the conclusions of this article is included within the article.

## References

[1] WHO Classification of Tumours Editorial Board. Soft tissue and bone tumours. 5th ed. Lyon: International Agency for Research on Cancer; 2020.

[2] Schneller J. Multifocal glomangiomyomas in the chest wall of a young man. Arch Pathol Lab Med 2001; 125: 1146–7. 11520261 10.5858/2001-125-1146-MGITCW

[3] Tsuruta Y, Mori T, Yoshioka M, et al. A case of glomus tumor in chest wall. J Jpn Assoc Chest Surg 2003; 17: 107–11. (in Japanese)

[4] Neelaiah S, Suryanarayanarao V. Glomus tumour of the chest wall. Case Rep Clin Pract Rev 2005; 6: 278–80.

[5] Uchiyama M, Kato T, Kunitani K, et al. Multiple glomus tumors in chest wall and buttocks. Kyobu Geka 2011; 64: 116–9. (in Japanese) 21387615

[6] Venugopal PR. Extradigital glomus tumor-a rare cause for undiagnosed chronic pain in unusual sites. Indian J Surg 2015; 77(Suppl 3): 910–2. 27011481 10.1007/s12262-014-1062-1 PMC4775574

[7] Temiz G, Şirinoğlu H, Demirel H, et al. Extradigital glomus tumor revisited: painful subcutaneous nodules located in various parts of the body. Indian J Dermatol 2016; 61: 118. 10.4103/0019-5154.174080 PMC4763633 26955123

[8] Yim IH, Will MB, Carnochan FM, et al. A glomus tumor with recurrence and malignant transformation in the chest wall: a cautionary tale of seeding? Ann Thorac Surg 2016; 102: e397–9. 27772590 10.1016/j.athoracsur.2016.04.052

[9] Kambhampati S, Kambhampati A. Subcutaneous glomus tumor of chest wall. Indian J Case Rep 2018; 4: 481–2.

[10] Zanjani LO, Shafiee Nia B, Vosoughi F, et al. An unusual case of chest wall glomus tumor presenting with axillary pain: a case report and literature review. Eur J Med Res 2021; 26: 49. 34034818 10.1186/s40001-021-00518-6 PMC8146208

[11] Alyaseen HN, Al Ghadeer HA, Al-Ghanim ME, et al. Extradigital glomangioma of the cutaneous chest wall. Cureus 2021; 13: e17910. 34660105 10.7759/cureus.17910 PMC8509110

[12] Bhalchandra Londhe S, Anchan C, Mahajan P. Extra digital glomus tumor causing scapulothoracic impingement: a case report. J Clin Orthop Trauma 2021; 22: 101595. 34589410 10.1016/j.jcot.2021.101595 PMC8453188

[13] Engle JA, Dibb JT, Kundu S, et al. Glomus tumor of the chest wall with metastases to lung. Cureus 2024; 16: e69122. 39398735 10.7759/cureus.69122 PMC11466728

[14] Jagelavicius Z, Mickeviciute E, Zurauskas E, et al. Primary glomangioma of the chest wall: case report and review of literature. J Surg Case Rep 2025; 2025: rjaf300. 40357460 10.1093/jscr/rjaf300 PMC12066395

[15] Babeau F, Knafo S, Rigau V, et al. Paravertebral glomangioma mimicking a schwannoma. Neurochirurgie 2013; 59: 187–90. 24367799 10.1016/j.neuchi.2013.06.002

[16] Folpe AL, Fanburg-Smith JC, Miettinen M, et al. Atypical and malignant glomus tumors: analysis of 52 cases, with a proposal for the reclassification of glomus tumors. Am J Surg Pathol 2001; 25: 1–12. 11145243 10.1097/00000478-200101000-00001

[17] Gombos Z, Zhang PJ. Glomus tumor. Arch Pathol Lab Med 2008; 132: 1448–52. 18788860 10.5858/2008-132-1448-GT

[18] Hazani R, Houle JM, Kasdan ML, et al. Glomus tumors of the hand. Eplasty 2008; 8: e48. 18997858 PMC2567120

